# Primary cerebral malignant melanoma: A case report with literature review

**DOI:** 10.1097/MD.0000000000005805

**Published:** 2017-01-27

**Authors:** Kai Tang, Xiangyi Kong, Gengsheng Mao, Ming Qiu, Haibo Zhu, Lei Zhou, Qingbin Nie, Yi Xu, Shiwei Du

**Affiliations:** a Department of Neurosurgery, Beijing Tiantan Hospital, Capital Medical University; b Department of Neurosurgery, Peking Union Medical College Hospital, Chinese Academy of Medical Sciences, Dongcheng District, Beijing, P. R. China; c Department of Anesthesia, Critical Care and Pain Medicine, Massachusetts General Hospital, Harvard Medical School, Harvard University, Boston, MA; d Department of Neurovascular Surgery, Chinese Armed Police General Hospital, Haidian District; e Department of Neurosurgery, Xuanwu Hospital, Capital Medical University, Xuanwu District, Beijing, P. R. China.

**Keywords:** case presentation, differential diagnosis, primary cerebral malignant melanoma

## Abstract

Primary intracranial melanomas are uncommon and constitute approximately 1% of all melanoma cases and 0.07% of all brain tumors. In nature, these primary melanomas are very aggressive and can spread to other organs.

We report an uncommon case of primary cerebral malignant melanoma—a challenging diagnosis guided by clinical presentations, radiological features, and surgical biopsy results, aiming to emphasize the importance of considering primary melanoma when making differential diagnoses of intracranial lesions.

We present a rare case of a primary cerebral melanoma in the left temporal lobe. The mass appeared iso-hypodense on brain computed tomography (CT), short signal on T1-weighted magnetic resonance images (T1WI) and long signal on T2WI. It was not easy to make an accurate diagnosis before surgery. We showed the patient's disease course and reviewed related literatures, for readers’ reference. Written informed consent was obtained from the patient for publication of this case report and any accompanying images. Because of this, there is no need to conduct special ethic review and the ethical approval is not necessary.

After surgery, the pathological examination confirmed the diagnosis of melanoma. The patient was discharged without any complications and went on to receive adjuvant radiochemotherapy.

It is difficult to diagnose primary cerebral melanoma in the absence of any cutaneous melanosis. A high index of clinical suspicion along with good pathology reporting is the key in diagnosing these extremely rare tumors.

## 1 Introduction

In human beings, melanocyte exists in the uvea, cerebral parenchyma, leptomeninges, mucous membranes, and skin.^[1]^ Malignant melanomas could originated from melanocytes, most of which are of cutaneous origins. Primary cerebral melanomas, derived from the melanocytic element normally present in the leptomeninges, are rare and occur in around 1% of all melanoma cases.^[2,3]^ Although preoperative anamnesis and computed tomography (CT) patterns are very similar to the presences of meningiomas or benign melanocytomas, these tumors show variable prognosis that depends on tumor site and extent of resection; therefore, to make an accurate diagnosis and identify the exact extent of the tumor area is important.^[1]^ Most patients with cerebral melanomas show clinical manifestation of increased intracerebral pressure (43%), neurological dysfunctions (35%), convulsion or subarachnoid hemorrhage (16%). Primary cerebral melanoma has a male dominance.^[3]^

Since melanocytes exist in normal leptomeningeal tissues, it is not unexpected that primary melanomas could develop within the central nervous system (CNS). Nevertheless, mostly, melanomas involving CNS represent metastases. Primary cerebral melanomas were not reported commonly. They can spread to other systems and organs and are aggressive in nature. It is commonly a diagnosis of exclusion of metastatic disease based on an oral or skin or uveal melanoma by appropriate evaluations as brains are the most common sites for metastases from melanoma lung or breast. Generally, solitary intracranial lesions with no melanomatous lesions found outside the CNS could be primary melanomas. In order to achieve a desirable survival, the key treatment of choice for local intraparenchymal melanomas is complete resection plus radiochemotherapy.^[4]^ Temozolomide, as an effective chemotherapeutic medication, can cross blood brain barriers and exert cytotoxic effects on cancer cells in patients with malignant melanoma.^[4]^ However once there are cancer spreads to leptomeninges, the overall median survival is generally only 10 weeks. We herein reported a case of the primary cerebral melanoma in the left temporal lobe, which was confirmed by histopathological examination of the excised mass. Also, related literatures were reviewed.

## 2 Case presentation

A 35-year-old woman presented with complaints of headache, nausea, and vomiting for 2 weeks. Her neurological examination was normal. He had no special previous history or family history. The results of routine blood chemical analysis and a complete blood count were normal.

**Figure 1:**
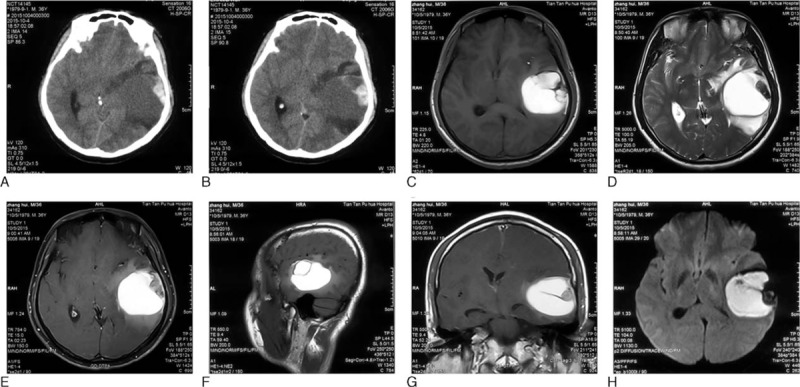
Preoperative brain CT and MRI images. (A and B) Axial view, CT images. (C) Axial view, T1-weighed image (T1WI). (D) Axial view, T2WI. (E) Axial view, contrast-enhanced T1WI. (F) Sagittal view, T1WI. (G) Coronal view, contrast-enhanced T1WI. (H) Axial view, diffusion weighted imaging (DWI). The mass appeared iso-hypodense on brain computed tomography (CT), short signal on T1-weighted magnetic resonance images (T1WI) and long signal on T2WI. It was not easy to make an accurate diagnosis before surgery.

CT scan showed a dural-based, extraaxial, well-defined, iso-hypodense area measuring around 5.1 cm × 4.2 cm × 3.5 cm (proved to be sludged blood by pathological examination afterwards) with an inside oval hyperdense lesion near the lateral border adjacent to the skull (proved to be a cystic lesion postoperatively, Fig. 1A and B). Irregular peritumoral vasogenic brain edema (hypodense) could be seen. Cerebral magnetic resonance imaging (MRI) with contrast revealed a mass in the left temporal area whose signal was short on T1-weighted image (T1WI) and long on T2WI. The peripheral cystic part mentioned above showed iso-long signal on T1WI and iso-short signal on T2WI, and was slightly enhanced after administration of gadolinium. The pathological field was surrounded by irregular edema (Fig. 1C–H). According to the CT and MRI features, a meningioma with intratumoral bleedings or a hemorrhagic metastasis was suspected.

**Figure 2:**
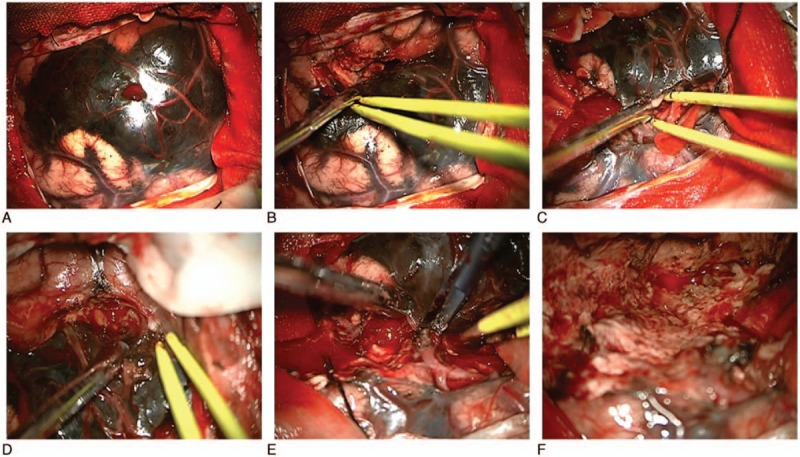
Intraoperative pictures showing the tumor was grossly removed completely. Although the tumor was adherent to the arachnoid membrane and bled easily, the main tumor was separable from the brain parenchyma and grossly totally removed.

A left frontotemporal craniotomy was performed, and at the dural opening, a large, blackish, vascular, firm tumor with multiple small satellite lesions in the arachnoid membrane was observed (Fig. 2A) and the main tumor was found to be embedded in the left temporal lobe along the sylvian fissure, with the brain acquiring a dark black coloration. Although the tumor was adherent to the arachnoid membrane and bled easily, the main tumor was separable from the brain parenchyma and grossly totally removed (Fig. 2B–F). Cerebrospinal fluid examinations did not reveal any evidences of malignant or atypical cells.

**Figure 3:**
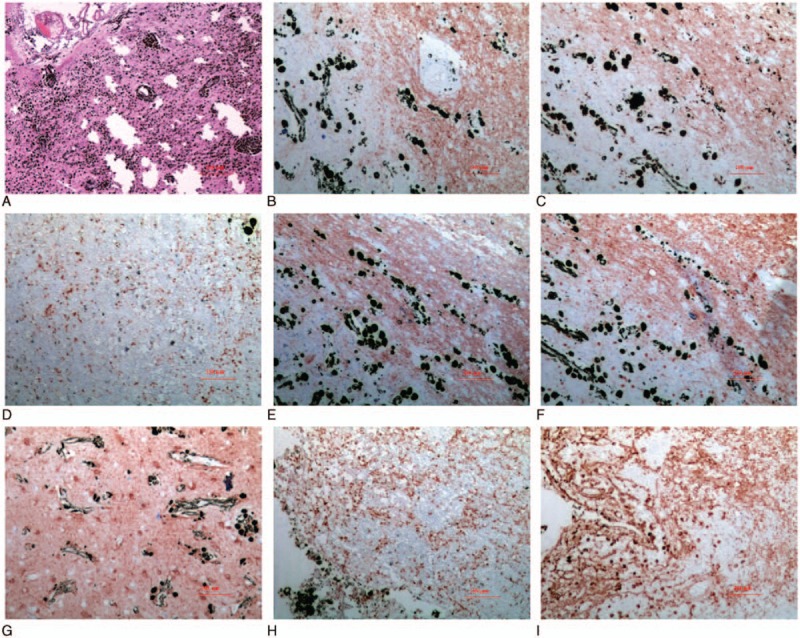
Histopathological and immunohistochemical (IHC) examinations of the postsurgical specimen. (A) Hematein–Eosin staining. (B) HMB-45. (C) CD68. (D) Ki-67. (E) Melan A. (F) Oligo-2. (G) S-100 protein. (H) VEGF. (I) Vimentin.

Pathohistological examinations confirmed the large part of sludged blood and the small part of cystic component, and revealed sheets of heavily pigmented cells with large nuclei and prominent nucleoli, consistent with malignant melanoma. The tumor was directly attached to the cerebral cortex but did not penetrate into the brain tissue. Immunohistochemistry showed a positive reaction for antimelanoma antibody (HMB-45), Melan A, S-100 protein, vascular endothelial growth factor (VEGF), glial fibrillary acidic protein (GFAP) and Vimentin, a partly positive reaction for oligodendrocyte transcription factor 2 (Olig 2) and CD68, and negative for cytokeratin (CK). The marker of proliferation Ki-67 was 3% positive staining. These findings were suggestive of a cerebral melanoma (Fig. 3).

Subsequent examinations, including dermatological physical examination and ophthalmologic fundoscopic examination, endoscopy of the gastrointestinal tract, body CT, and the positron emission tomography (PET) scan of the whole brain and body revealed no evidence of melanoma in other parts of the body.

**Figure 4:**
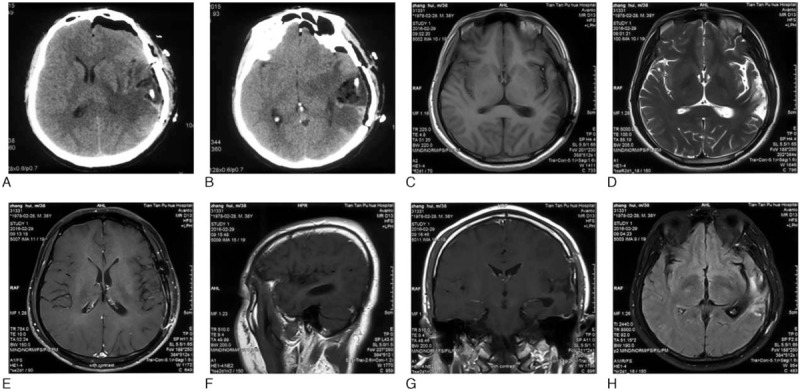
Postoperative brain CT and MRI images. (A and B) Axial view, CT images 1 day after surgery. (C–H) Nearly 5 months after surgery. (C) Axial view, T1-weighed image (T1WI). (D) Axial view, T2WI. (E) Axial view, contrast-enhanced T1WI. (F) Sagittal view, T1WI. (G) Coronal view, contrast-enhanced T1WI. (H) Axial view, diffusion weighted imaging (DWI).

The patient was discharged 5 days postoperatively without any complications and went on to receive adjuvant radiochemotherapy (concomitant stereotactic radiosurgery + temozolomide). For more than 11 months of follow-up, the patient recovered well without relevant sequelae or recurrences (Fig. 4).

## 3 Discussion

**Figure 5:**
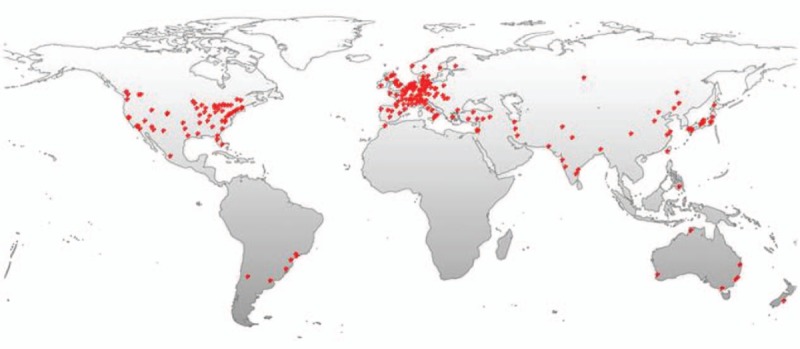
A world map with the global distribution of intracranial melanoma related publications based on the analysis of their geolocational data.

Melanomas originate from melanocytes, existing in the human uvea, cerebral parenchyma, leptomeninges, mucous membranes, and skin.^[5,6]^ Intracranial melanomas can be divided into metastatic type or primary type, and primary cerebral melanoma accounts for 1% of all melanomas.^[7]^ The literature quantity related to intracranial melanomas shows a tendency to increase year by year generally. On the basis of a world map for global distributions of intracranial-melanoma-related literatures according to the geolocational data analyses, the areas that the literatures come from concentrated mainly on Japan, Eastern United States and Europe (Fig. 5). Primary cerebral melanomas mainly derive from leptomeningeal melanocytes around the cervical cord or in the skull base. Metastatic melanomas presents mainly the features as followings: develop mainly in the elderly, whilst primary cerebral melanomas often develop in patients under 50 years of age and uncommonly metastasize to systemic organs; a poor and rapid clinical course because of possible systemic metastases; and multiple intracerebral tumors.^[8]^ Somers and his colleagues put forward that intracranial melanomas are more likely to be primary if there are no evidences of melanomas outside the CNS.^[9]^ Primary intracranial melanomas are classified as 4 different categories: melanosis of brain/spinal cord meninges correlated to cutaneous pigmentations (phakomatosis or neurocutaneous melanoses), primary cerebral isolated melanoma, discrete spinal-cord melanoma, and diffuse leptomeningeal melanomatoses.^[10]^ Our patient had no systemic melanomas detected by clinical examinations. Instead, only a solitary isolated mass was found. Regarding primary cerebral melanomas, although origins of melanin cells are still not fully unveiled, some histogenetic hypotheses were put forward by scholars (Table 1).^[11]^

**Table 1:**
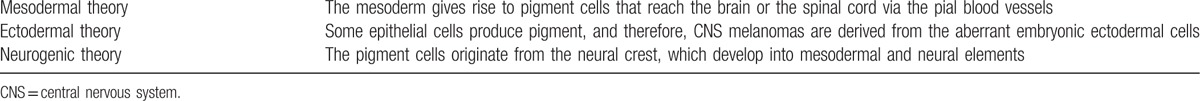
Several histogenetic theories regarding primary cerebral melanomas.

Suranagi et al^[12]^ once reviewed 19 cases reported over a period of 25 years. Male predominance was noted. The tumor was frequently observed in the middle age to elderly age group. Most of the cases involved different areas of the cerebrum except for 3 cases occurring in the cerebellopontine angle (CPA). The presenting symptoms were headache, ocular symptoms, hemiparesis, and seizures. Six patients showed recurrence mostly within 18 months, except in 1 case where the tumor recurred after 23 years. Excision of the tumor was possible in a total of 14 cases; while others were given adjuvant chemotherapy and radiotherapy. Five patients died within 1-year, while 1 patient survived for 17 years. Extension with metastasis was present in 3 cases. Our case also received chemotherapy or radiotherapy and did not show recurrence or metastasis at 8-month follow-up.

The preoperative diagnoses of primary cerebral melanomas are usually difficult, except in cases related to neurocutaneous melanosis or if containing cells containing melanin could be found in cerebrospinal fluid.^[13]^ The CT imagings of cerebral melanomas are not specific. Relatively typical CT findings of primary solitary cerebral melanomas include a superficially located hyperdense mass that is enhanced dramatically after contrast enhancement. The increased attenuation on the precontrast CT scan is thought to be caused by intratumoral hemorrhage and/or the presence of melanin. These findings may suggest the diagnosis of melanoma rather than glioma but often mimic the presence of meningioma.^[14]^ As for our case, the disease on CT presented as a general iso-hypodense area (old sludged blood) with a small, hyperdense cystic lesion near the lateral edge adjacent to the skull and an irregular hypodense peritumoral brain edema belt, which was different from the majority of cases reported before.

The MRI imaging feature varies a lot, depending on the types of melanomas and the absence or presence of intratumoral hemorrhages.^[15]^ In typical melanotic melanomas, the melanin has paramagnetic effects which derive from the presences of stable organic-free radicals inside. The unpaired electrons of these free radicals interact with the water protons, resulting in shortening of both T1 and T2 relaxation times and producing short signal on T1WI and short signal on T2WI.^[16]^ Intratumoral hemorrhage produces a heterogeneous signal on T1WI and T2WI. Amelanotic melanoma and melanoma without a hemorrhagic component tend to appear iso-long signal on T1WI and moderately long signal on T2WI.^[17]^ Most of the lesion of our case was old intratumoral hemorrhage, appearing on MRI short T1 and long T2 signals, which was also somewhat different from previous cases.

Metastases from melanomas of a cutaneous origin should be considered firstly when making a differential diagnosis.^[18]^ Primary tumors are known in approximately 90% of patients carrying secondary tumors, 45% of which have CT/MRI evidences of multiple masses; but the primary tumors are unknown in 10% of these cases.^[19]^ At times, primary melanomas are only discovered upon clinical examinations after histopathological recognitions of metastases. Also, primary intracranial melanomas are different from metastases in symptom durations and life expectancy: the median survivals for melanoma metastases are only 5 to 6 months whilst primary melanoma patients can commonly survive over 17 years.^[19]^ Other differential diagnoses are listed in Table 2.

**Table 2:**
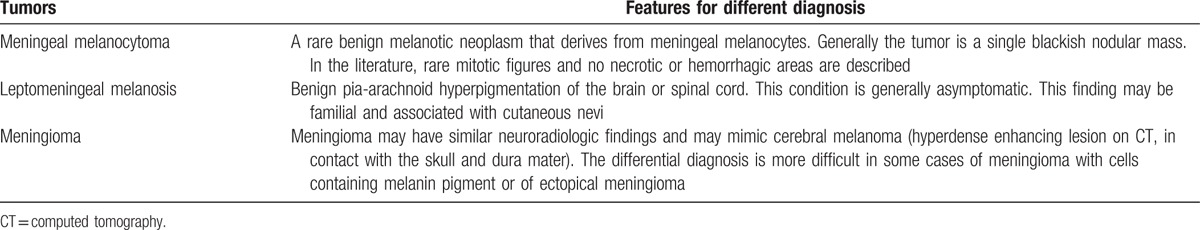
Other differential diagnoses besides metastatic cerebral melanomas.

Primary cerebral melanomas are supposed to be treated by complete resections plus effective postoperative radiochemotherapy.^[17,18]^ In recent years, the most effective and common chemotherapy drug is dacarbazine, which could be used after operation or radiotherapy, presenting the effectiveness rate of 16% to 20%.^[3]^ Previously, some researches indicated that stereotaxic radiosurgery (SRS), alone or plus whole-brain radiotherapy, might improve lifespans of cerebral melanoma patients.^[20]^ Salpietro et al^[21]^ put forward that specific immunotherapies were crucial adjuvant methods in treating small residual malignant melanomas, and presented low toxicities. High doses of interferon (IFN) β or IFN α-2b were found to be able to improve survival time and disease control, but the optimal dosages are still controversial. Hunder et al^[22]^ reported the bioremediation encountered by a patient (in remission for 2 years) with multiple melanoma metastases, but did not show intracranial metastatic lesions. With adjuvant radiotherapy and chemotherapy, the present patient is tumor free 8 months later and is still under follow-up.^[23]^

## 4 Conclusion

It is difficult to diagnose primary cerebral melanoma in the absence of any cutaneous melanosis. A high index of clinical suspicion along with good pathology reporting is the key in diagnosing these extremely rare tumors. The prognoses are disable in cases of totally resected melanomas. The roles of adjuvant radiochemotherapy have not been well-established. Improvements in targeted therapies, immunotherapies, and chemotherapies might provide more efficient treatments for primary cerebral melanomas.
